# Plasmons in Finite Spherical Electrolyte Systems: RPA Effective Jellium Model for Ionic Plasma Excitations

**DOI:** 10.1007/s11468-015-0064-6

**Published:** 2015-10-05

**Authors:** Witold Aleksander Jacak

**Affiliations:** Department of Quantum Technology, Wrocław University of Technology, Wyb. Wyspiańskiego 27, 50-370 Wrocław, Poland

**Keywords:** Surface plasmons, Soft plasmonics, Damping of ion plasmons

## Abstract

Plasmons are fundamental collective excitations in many particle charged systems like in free electron liquid in metals, high energy nuclear plasma in solar core or in fusion devices, in ion gas in ionosphere or in intra- and inter-galactic gas clouds. Plasmons play a central role also in small systems, in particular in metallic nanoparticles and in their arrays allowing for subdiffraction light manipulation. In analogy to metallic nanoparticles, we have developed description of the soft plasmonics in finite electrolyte systems confined in micrometer scale by insulating membranes. Plasmon-type excitations in such finite ionic systems are determined via originally formulated theoretical model allowing to describe surface and volume plasmons in confined geometry of the ion liquid. Size-effect for attenuation of surface plasmons in the finite electrolyte system is described and its various regimes are identified. The cross-over in the plasmon damping system-size-dependence is demonstrated including scattering of ions and their energy losses via irradiation. The plasmon resonances in ion systems replicate the metal cluster plasmon phenomena, though in distinct energy and size scale related to larger ion mass and lower ion concentration (in low energy plasma) in comparison to electrons in metals. The possibility for tuning plasmon resonances in finite ionic systems in a wide range by changing system size, ion, and electrolyte parameters is demonstrated.

## Introduction

Recent experimental and theoretical investigations of plasmon oscillations in metallic nanoparticles have focused attention on their fundamental character and numerous applications. In particular, the so-called plasmon effect in solar cells modified on the nanoscale with surface-deposited metallic particles has led to improvements of their efficiency [1–6]. The surface plasmon oscillations in these particles play a mediating role in harvesting energy from sunlight because of the particle’s radiative properties. The radiated energy from plasmon oscillations is preferable for transport applications. As was observed experimentally and predicted theoretically, irradiation losses of plasmon energy are strongly sensitive to the size of metallic nanoparticles [7, 8].

The strong irradiation of plasmon oscillations in metallic nanoparticles also plays a major role in the construction of plasmonic waveguides with high transference efficiency. Several experimental studies [9, 10] have indicated that periodic linear structures of metallic nanoparticles serve as efficient plasmon waveguides with low damping [11–13]. The wavelengths of plasmon polaritons propagating in such structures are typically shorter than wavelengths of light with the same frequency by one or two orders of magnitude, enabling avoidance of diffraction limits in light circuits [14–16]. This avoidance enables construction of plasmonic opto-electronic nanodevices not available when using only light waveguides limited by diffraction constraints. The efficient energy transfer in plasmonic waveguides is also supported by the radiative properties of surface plasmons in metallic nanocomponents.

Accelerating charges irradiate electromagnetic waves, and the related energy loss can be accounted for by an effective electric field that hampers charge movement called as the Lorentz friction [17, 18]. In the case of an oscillating dipole such as the dipole-type surface plasmons in a metallic cluster, the Lorentz friction force is proportional to the third-order time-derivative of the dipole [17]. Let us emphasize here that the strong irradiation of surface plasmons in metallic clusters is exclusively present in sufficiently large metallic particles (larger than ca. 15 nm in diameter, for Au or Ag). Ultra-small metallic clusters with diameters of 2–10 nm do not exhibit irradiation efficiency as high as that of nanospheres with radii *a*>15 nm, mostly because of the much lower number of electrons in so small clusters in comparison to the number of electrons in larger nanospheres. In particular, extensive attention has been focused on large nanoparticles of noble metals (gold, silver, and copper) because their plasmon resonances are located within the visible region of the electromagnetic spectrum.

Plasmons in metallic nanostructures focused interest on similar local charge density oscillations in amorphous materials or in other unconventional systems [19–21] including also ionic systems and interaction of ions with metallic plasmons [22] as well as soft flexible photonic crystals with wide applications [23]. Combining metallic nanoparticles with host materials whose dielectric properties can be tuned by means of an external control is one route to create active plasmonics. By exploiting the outstanding properties of self-organizing materials, so-called smart matter, including plasmons interacting with light, a bridge between two branches of physics: ‘hard matter’ and ‘soft matter’ can be built. The soft plasmonics and plasmonic crystals have found already many applications in photonics and in sensing [24].

Some examples of natural and man-made plasma besides of the metal-electron-plasma can be listed as follows: laboratory gas discharge, e.g., in vacuum tubes, spark gaps, welding arcs, and neon or fluorescent lights, controlled thermonuclear fusion experiments, e.g., in tokamak, earth ionosphere that is partially photo-ionized by solar ultra-violet radiation, sun’s core, where fusion of hydrogen to form helium generates the sun’s heat, solar wind, i.e., the wind of plasma that blows off the sun, interstellar, and intergalactic ionic gas medium. The plasmon oscillations—local fluctuations of the charge density—have the frequency proportional to the square root of the charge carrier density and this frequency varies between 10^18^ 1/s (for solar core), across 10^8^ 1/s (ionosphere) to 10^2−4^ 1/s (interstellar and intergalactic ionized gas). The plasmon excitations in the ionosphere have, in particular, a great importance in radio-communication and in over-horizon radar techniques. Much attention, also in experiments with ionized fullerene gas, has been recently paid to electrostatic waves in so-called pair-ion-plasma consisting of only positive- and negative-charged equal mass particles with a time-space parity kept because the mobility of the equal mass particles in electromagnetic fields is the same [25, 26]. The magneto-hydrodynamics of such a system may have importance in understanding of cosmic ion systems in electromagnetic field and in fusion plasma, previously analyzed upon the Vlasov kinetic equation [27]. Despite the ionic plasmons are thus well recognized in various large and open systems [28, 29], their counterparts in small confined electrolytes are not penetrated as of yet, but probably offer a rich physics as they might replicate the plasmonics in small metallic particles. Micrometer scale of electrolyte confinement is frequent in bio-cell organization, where a cytoplasm containing various ions is separated from surroundings by lipid cellular membrane. The local charge fluctuations in such ionic finite systems can be important in biophysical phenomena of communication and signaling as well as in local energy transport. An example is the newly developed plasmon-polariton model of the so-called saltatory-conduction in neuron long axon [30].

An interesting question arises as to whether similar to complicated metallic plasmonic effects are possible with ionic carriers instead of electrons. Many finite ionic systems in the form of electrolyte enclosed by membranes are found in biological structures. The question then arises as to what role plasmonic phenomena would play in such structures and whether the radiative properties of plasmon fluctuations would be as significant in ionic systems as in metals. One can reasonably expect that ionic plasmon effects would be located in different energy and wavelength scale regions compared to those of metallic systems due to the larger mass of ions and smaller concentration than for metal electrons. The ionic *soft plasmonics* could be linked to the functionality of biological systems in which electricity is of an ionic rather than an electronic character, such as cell signaling, membrane transfer, and nerve-cell conductivity.

Ionic systems are much more complicated in comparison to a metal crystal structure with free electrons. Therefore, identification of appropriate model simplifications is of primary significance to properly describe collective charge excitations in electrolytes along with keeping analogy with metal plasmonics.

In the present paper, we will consider a finite spherical ionic system (e.g., liquid electrolyte confined within a spherical membrane) to identify plasmonic excitation. We will determine their energies with respect to various parameters of the ionic system, with special attention paid to the irradiation properties of ionic plasmons.

The paper is organized as follows. In the first paragraph, the effective model for plasmon fluctuations in finite spherical binary electrolyte system is formulated via introducing of specially defined two ion-jellium components (for electrolytes, the jellium is fictitious and an auxiliary model construction unlike to metals where the jellium is the real rigid crystal positive core). The model is utilized in the next paragraph to identify surface and volume ionic plasmon excitations in finite spherical electrolyte system ranged by neutral dielectric membrane. Both self-frequencies and the attenuation rates are next estimated for ionic surface and volume plasmons in the considered system, for various ion and electrolyte concentration parameters and system sizes. The radiative properties of the ion surface dipole plasmons are next examined in details which are of importance for the energy and information transfer in ionic systems in possible application to electrolyte finite components in biological cellular structures and their electrical functioning.

## Fluctuations of Charge Density in a Finite Spherical Ionic System

For a simple two-component ionic system, we address the water solution of ions of both signs, creating an electrolyte with balanced total charge, enclosed in finite size spherical volume ranged by electrically neutral insulating membrane. At equilibrium, also local charge cancellation holds. Both types of ionic carriers can form, however, density fluctuations, resulting in disruption of the local electric equilibrium. The total charge conservation and neutrality condition require that any density fluctuation of negative charges must be accompanied by equivalent fluctuation in the positive ions, possibly in another even distant place of the system, and vice versa. Therefore, we effectively deal with density fluctuations of ions, positive and negative (but always mutually compensated), with respect to a uniform fictitious background charge distributions of the opposite sign in some analogy to the jellium model in metals. In the case of two component electrolyte, these auxiliary uniform background charge distributions cancel mutually themselves and do not modify the system and its energy. Each of these auxiliary ionic jelliums has the total charge equaled to the sum of charges of all ions of the opposite sign. Thus, the opposite oscillations of both types of ions are represented here as the equivalent sum of two ion-jellium systems also simultaneously oscillating.

### Definition of the Model

To develop a model according to these guidelines, let us consider a spherical system with a radius *a* and a balanced total charge of both sign ions with uniform equilibrium density distributions *n*^+(−)^(**r**) = *n*Θ(*a*−*r*) (where Θ(*r*) is the Heaviside step function). The equilibrium density of the charged liquid, denoted by *n*, will be treated as a parameter and *n* = *ηN*_0_ will be assumed, where *η* is the molarity of the electrolyte within the sphere and *N*_0_ is the one-molar electrolyte concentration of ions.

The Hamiltonian for the simplest two-component ion system has the form,

1 $$\begin{array}{@{}rcl@{}} \hat{H}_{ion}& =& -\sum\limits_{i =1}^{N^{-}}\frac{\hbar^{2} {\nabla_{i}^{2}}}{2m^{-}} - \sum\limits_{j =1}^{N^{+}}\frac{\hbar^{2} {\nabla_{j}^{2}}}{2m^{+}} - \sum\limits_{i,j}^{N^{-},N^{+}} \frac{q^{-}q^{+} }{ \varepsilon |\mathbf{r}_{i} - \mathbf{r}_{j}|}\\ && +\frac{1}{2}\sum\limits_{i,i^{\prime},i\neq i^{\prime}}^{N^{-}}\frac{(q^{-})^{2}}{\varepsilon |\mathbf{r}_{i}-\mathbf{r}_{i^{\prime}}|} +\frac{1}{2}\sum\limits_{j,j^{\prime}j\neq j^{\prime}}^{N^{+}}\frac{(q^{+})^{2}}{\varepsilon |\mathbf{r}_{j}-\mathbf{r}_{j^{\prime}}|}, \end{array} $$

where *q*^−(+)^, *m*^−(+)^, and *N*^−(+)^ are the charge, mass, and total number of the −(+) ions, respectively. Indices *i* and *j* are introduced to distinguish two sorts of ions. To analyze this complicated system, we propose the following approximation. Assume, for simplicity, *q*^−^ = −*q*^+^ = *q*, *N*^−^ = *N*^+^ = *N*, and *m*^−^ = *m*^+^ = *m* (the generalization to distinct charges and masses of both sign ions is straightforward), and let us add and subtract the same terms (the last four terms in the following expression for the Hamiltonian),

2 $$\begin{array}{@{}rcl@{}} \hat{H}_{ion}& =& -\sum\limits_{i =1}^{N}\frac{\hbar^{2} {\nabla_{i}^{2}}}{2m} - \sum\limits_{j =1}^{N}\frac{\hbar^{2} {\nabla_{j}^{2}}}{2m} - \sum\limits_{i,j} \frac{q^{2} }{ \varepsilon |\mathbf{r}_{i} - \mathbf{r}_{j}|}\\ &&+\frac{1}{2}\sum\limits_{i,i^{\prime},i\neq i^{\prime}}^{N}\frac{q^{2}}{\varepsilon |\mathbf{r}_{i}-\mathbf{r}_{i^{\prime}}|} +\frac{1}{2}\sum\limits_{j,j^{\prime}j\neq j^{\prime}}^{N}\frac{q^{2}}{\varepsilon |\mathbf{r}_{j}-\mathbf{r}_{j^{\prime}}|}\\ &&-q^{2}\sum\limits_{j} \int\frac{n (\mathbf{r})d^{3} \mathbf{r}}{\varepsilon|\mathbf{r}_{j}-\mathbf{r}|}\\ &&-q^{2}\sum\limits_{i} \int\frac{n (\mathbf{r})d^{3} \mathbf{r}}{\varepsilon|\mathbf{r}_{i}-\mathbf{r}|} +q^{2}\sum\limits_{j} \int\frac{n (\mathbf{r})d^{3} \mathbf{r}}{\varepsilon|\mathbf{r}_{j}-\mathbf{r}|}\\ &&+q^{2}\sum\limits_{i} \int\frac{n (\mathbf{r})d^{3} \mathbf{r}}{\varepsilon|\mathbf{r}_{i}-\mathbf{r}|}. \end{array} $$

In this way, we have formally introduced a jellium of spherical shape for both types of ions, with the density *n* ideally compensating opposite charges of uniformly distributed ions, *n*(**r**) = *n*Θ(*a*−*r*), *a* is the sphere radius. Assuming now, upon a rough approximation that,

3 $$ q^{2}\sum\limits_{j} \int\frac{n (\mathbf{r})d^{3} \mathbf{r}}{\varepsilon|\mathbf{r}_{j}-\mathbf{r}|} +q^{2}\sum\limits_{i} \int\frac{n (\mathbf{r})d^{3} \mathbf{r}}{\varepsilon|\mathbf{r}_{i}-\mathbf{r}|}- \sum\limits_{i,j} \frac{q^{2} }{ \varepsilon |\mathbf{r}_{i} - \mathbf{r}_{j}|}\simeq 0,  $$

one can can separate the Hamiltonian (1) into the sum $ \hat {H}_{ions}=\hat {H}^{-}+\hat {H}^{+} $, where

4 $$\begin{array}{@{}rcl@{}} \hat{H}^{-(+)} &=& \sum\limits_{j } \left[ -\frac{\hbar^{2} {\nabla_{j}^{2}}}{2m} -q^{2} \int\frac{n (\mathbf{r})d^{3} \mathbf{r}}{\varepsilon |\mathbf{r}_{j} - \mathbf{r}|} \right] \\&&+\frac{1}{2}\sum\limits_{j\neq j^{\prime}}\frac{q^{2}}{\varepsilon|\mathbf{r}_{j}-\mathbf{r}_{j^{\prime}}|}. \end{array} $$

The latter term in the right-hand side of Eq. 4 corresponds to the interaction between ions of the same sign, whereas the second term in the first sum describes the interaction of these ions with the jelliums of opposite sign (*ε* is the dielectric constant of the electrolyte medium). Because of the separation of the Hamiltonian (1), one can consider a single Hamiltonian (4).

The question is the applicability of the condition (3). This condition means that the mutual interaction between two sign ion fluctuations is equal to the sum of interactions of these fluctuations with the fictitious jelliums of opposite signs ideally neutralized themselves. For small fluctuations, one can argue that the total energy of the ion interaction is not changed by the approximation (3) and it may be used to the assessment of energy scale of ion fluctuations. The advantage of such an approach is the close analogy to the description of plasmons in metals, including the direct definition of the shape of the system with the explicit rigid jellium form. Let us emphasize that the apparent decoupling of both sign ion fluctuations via Eq. 3 is not complete in fact. Each fluctuation of ion density beyond the uniform equilibrium distribution, let say of negative ions, produces noncompensated positive charging of the fictitious jellium being in fact the fluctuation of positive ions in the real system. The decoupling corresponds thus to duplication of the ion-jellium dynamics description for both ion types, without, however, the change of interaction energy, for small fluctuations at least. Both the ion-jellium dynamics describe the same fluctuations of ion densities in terms of opposite sign ions, which are actually coupled in the binary electrolyte. Such a picture is of particular usefulness for the case of the same charges and masses of ions, whereas it worsens with rising differences between ion parameters, when asymmetry between separate ion-jellium oscillations grows. The alternative way to introduce the jellium model for ions in the electrolyte might be the definition of an effective ion (with effective charge and mass in a far analogy to the two body problem) comprising all the two component dynamics described as fluctuations with respect to the single opposite sign jellium. Such a model would diminish, however, the level of degrees of freedom in comparison to the real system and in opposition to the two component ion-jellium model. Therefore, we will develop and test the two component ion-jellium model resulted from the approximate Eq. 3 aiming on even rough assessment of the energy scale of ion plasmon fluctuations.

Upon the proposed model, the equilibrium ion density determines the bulk plasmon frequency for the ion system (for each type of ions) according to the formula analogous to bulk metal [31], ${\omega _{p}^{2}}=\frac {4 \pi n q^{2}}{m}$, where *n* and *m* are the equilibrium uniform concentration and the mass of ions with charge *q*, respectively. Because *m* is larger than the electron mass, *m*_*e*_, and the ion concentration is usually smaller than that of electrons in metals, *ω*_*p*_ can be considerably reduced, even by several orders of magnitude. Noticeably, for electrons in metals, $\hbar \omega _{p} \simeq 10$ eV and typically falls in the ultraviolet region. In an ionic system, the plasmon frequency $\hbar \omega _{p}$ can be much lower: in the infrared or even lower-energy regions.

The form of the Hamiltonian (4) allows for its utilization in the scheme applied to electrons in metals [31–33]. A local density of ions can be written, analogous to the semiclassical Pines-Bhom random-phase approximation (RPA) of electrons in metals [31, 32], in the following form:

5 $$ \rho(\mathbf{r}, t)=<{\Psi}_{ion}(t)|\sum\limits_{j} \delta(\mathbf{r}-\mathbf{r}_{j}) |{\Psi}_{ion}(t)>, $$

where **r**_*j*_ denotes the coordinate of the *j*−*th* ion and the Dirac delta semiclassically fixes the *j*−*th* ion position; Ψ_*ion*_(*t*) denotes the ion wave-function corresponding to the Hamiltonian (4). The Fourier picture of the local density of ions has the form:

6 $$ \tilde{\rho}(\mathbf{k}, t)=\int \rho(\mathbf{r},t) e^{-i\mathbf{k}\cdot \mathbf{r}} d^{3} r = <{\Psi}_{ion}(t)|\hat{\rho}(\mathbf{k})|{\Psi}_{ion}(t)>, $$

where the operator $ \hat {\rho } (\mathbf {k})=\sum \limits _{j} e^{-i\mathbf {k}\cdot \mathbf {r}_{j}} $.

Using the aforementioned notation, one can rewrite $\hat {H}_{ion}$ in the following form, analogous to the case for metallic plasmons [31–33]:

7 $$\begin{array}{@{}rcl@{}} \hat{H}_{ion}& =& \sum\limits_{j =1}^{N} \left[ -\frac{\hbar^{2} {\nabla_{j}^{2}}}{2m}\right] - \frac{q^{\prime2}}{(2 \pi)^{3}} \int d^{3} k \tilde{n}(\mathbf{k}) \frac{2 \pi}{k^{2}} \left( \hat{\rho^{+}}(\mathbf{k}) + \hat{\rho}(\mathbf{k})\right)\\ && + \frac{q^{\prime2}}{(2 \pi)^{3}}\int d^{3} k \frac{2 \pi}{k^{2}}\left[ \hat{\rho^{+}}(\mathbf{k}) \hat{\rho}(\mathbf{k}) -N \right], \end{array} $$

where $ \tilde {n}(\mathbf {k})=\int d^{3} r n (\mathbf {r}) e^{-i\mathbf {k}\cdot \mathbf {r}}$ is the Fourier picture of the jellium distribution (in the derivation of Eq. 7 we have taken into account that $ \frac {4 \pi }{k^{2}}= \int d^{3} r \frac {1}{r} e^{-i\mathbf {k}\cdot \mathbf {r}}$), $q^{\prime 2}=\frac {q^{2}}{\varepsilon }$ .

Utilizing this form of the effective ion Hamiltonian, one can write out the dynamic equation in Heisenberg representation for the ion density fluctuations ([..] denotes the commutator),

8 $$ \frac{d^{2} \hat{\rho} (\mathbf{k}) }{dt^{2}}=\frac{1}{(i\hbar)^{2}} \left[\left[ \hat{\rho} (\mathbf{k}),\hat{H}_{ion} \right],\hat{H}_{ion} \right], $$

which attains the following form:

9 $$\begin{array}{@{}rcl@{}} \frac{d^{2} \delta \hat{\rho} (\mathbf{k}) }{dt^{2}}&=&-\sum\limits_{j}e^{-i\mathbf{k}\cdot \mathbf{r}_{j}}\left\{ -\frac{\hbar^{2}}{m^{2}}\left( \mathbf{k}\cdot \nabla_{j} \right)^{2} + \frac{\hbar^{2} k^{2}}{m^{2}}i \mathbf{k}\cdot \nabla_{j} +\frac{\hbar^{2} k^{4}}{4 m^{2}}\right\}\\ &&-\frac{4\pi q^{\prime2}}{m (2\pi )^{3}}\int d^{3}p \tilde{n} (\mathbf{k}-\mathbf{p})\frac{\mathbf{k}\cdot \mathbf{p}}{p^{2}} \delta \hat{\rho}(\mathbf{p})\\ &&-\frac{4\pi q^{\prime2}}{m (2\pi )^{3}}\int d^{3}p \delta \hat{\rho}(\mathbf{k}- \mathbf{p})\frac{\mathbf{k}\cdot \mathbf{p}}{p^{2}} \delta \hat{\rho}(\mathbf{p}). \end{array} $$

where $\delta \hat {\rho }(\mathbf {k}) = \hat {\rho }(\mathbf {k}) - \tilde {n} (\mathbf {k})$ describes the operator of local ion density fluctuations with respect to the equilibrium uniform density.

Averaging over the quantum states |Ψ_*ion*_>, we obtain the following equation for the ion density fluctuations: $\delta \tilde {\rho }(\mathbf {k},t)= <{\Psi }_{ion}|\delta \hat {\rho }(\mathbf {k},t)|{\Psi }_{ion}>= \tilde {\rho }(\mathbf {k},t) - \tilde {n} (\mathbf {k})$,

10 $$\begin{array}{@{}rcl@{}} \frac{\partial^{2} \delta \tilde{\rho} (\mathbf{k},t) }{\partial t^{2}}&=&-<{\Psi}_{ion}|\sum\limits_{j}e^{-i\mathbf{k}\cdot \mathbf{r}_{j}}\\&&\times\left\{ -\frac{\hbar^{2}}{m^{2}}\left( \mathbf{k}\cdot \nabla_{j} \right)^{2} + \frac{\hbar^{2} k^{2}}{m^{2}}i \mathbf{k}\cdot \nabla_{j} +\frac{\hbar^{2} k^{4}}{4 m^{2}}\right\}|{\Psi}_{ion}>\\ && -\frac{4\pi q^{\prime2}}{m (2\pi )^{3}}\int d^{3}p \tilde{n} (\mathbf{k}-\mathbf{p})\frac{\mathbf{k}\cdot \mathbf{p}}{p^{2}} \delta \tilde{\rho}(\mathbf{p},t)\\ && -\frac{4\pi q^{\prime2}}{m (2\pi )^{3}}\int d^{3}p \frac{\mathbf{k}\cdot \mathbf{p}}{p^{2}}\\ &&\times<{\Psi}_{ion}| \delta \hat{\rho}(\mathbf{k}- \mathbf{p}) \delta \hat{\rho}(\mathbf{p}) |{\Psi}_{ion}>. \end{array} $$

For small *k*, as with the semiclassical approximation for electrons [31, 33], the contributions of the second and third components of the first term on the right-hand side of Eq. 10 can be neglected as small in comparison to the first component (with the lowest power of *k*). The third term in the right-hand side of Eq. 10 is also small (and thus negligible), involving a product of two $ \delta \tilde {\rho }$ (which we assumed to be small, $ \delta \tilde {\rho }/n << 1$). This approach corresponds to the RPA formulated for bulk metal [31, 32]. Within the RPA, Eq. 10 takes the following shape:

11 $$\begin{array}{@{}rcl@{}} \frac{\partial^{2} \delta \tilde{\rho} (\mathbf{k},t) }{\partial t^{2}}&=&\frac{2 k^{2}}{3m} <{\Psi}_{ion}|\sum\limits_{j}e^{-i\mathbf{k}\cdot \mathbf{r}_{j}}\frac{\hbar^{2}{\nabla_{j}^{2}}}{2m}|{\Psi}_{ion}>\\ &&-\frac{4\pi q^{\prime2}}{m (2\pi )^{3}}\int d^{3}p \tilde{n} (\mathbf{k}-\mathbf{p})\frac{\mathbf{k}\cdot \mathbf{p}}{p^{2}} \delta \tilde{\rho}(\mathbf{p},t),\\ \end{array} $$

and, because of spherical symmetry,

$$\begin{array}{@{}rcl@{}} &&\!\!\!\!\!\!\!<{\Psi}_{ion}|\sum\limits_{j}e^{-i\mathbf{k}\cdot \mathbf{r}_{j}}\frac{\hbar^{2}}{m^{2}}\left( \mathbf{k}\cdot \nabla_{j} \right)^{2}|{\Psi}_{ion}>\\ &\simeq& \frac{2 k^{2}}{3m}<{\Psi}_{ion}|\sum\limits_{j}e^{-i\mathbf{k}\cdot \mathbf{r}_{j}}\frac{\hbar^{2}{\nabla_{j}^{2}}}{2m}|{\Psi}_{ion}>. \end{array} $$

Equation 11 can be rewritten in the position representation:

12 $$\begin{array}{@{}rcl@{}} \frac{\partial^{2} \delta \tilde{\rho} (\mathbf{r},t) }{\partial t^{2}}&=&-\frac{2 }{3m} \nabla^{2} <{\Psi}_{ion}|\sum\limits_{j}\delta(\mathbf{r}-\mathbf{r}_{j})\frac{\hbar^{2}{\nabla_{j}^{2}}}{2m}|{\Psi}_{ion}>\\ &&+\frac{{\omega_{p}^{2}}}{4\pi} \nabla \left\{ {\Theta}(a-r) \nabla \int d^{3}r_{1} \frac{1}{|\mathbf{r}-\mathbf{r}_{1}|} \delta \tilde{\rho}(\mathbf{r}_{1},t)\right\}. \end{array} $$

In the case of metals, the Thomas-Fermi formula is used to assess the averaged kinetic energy [32]:

13 $$\begin{array}{@{}rcl@{}} <{\Psi}_{ion}|-\sum\limits_{j}\delta(\mathbf{r}-\mathbf{r}_{j})\frac{\hbar^{2}{\nabla_{j}^{2}}}{2m}|{\Psi}_{ion}>\simeq \frac{3}{5} (3\pi^{2})^{2/3} \frac{\hbar^{2}}{2m} (\rho(\mathbf{r},t))^{5/3}\\ =\frac{3}{5} (3\pi^{2})^{2/3} \frac{\hbar^{2}}{2m}n^{5/3} {\Theta}(a-r)\left[1+\frac{5}{3}\frac{\delta \tilde{\rho}(\mathbf{r},t)}{n}+...\right].\\ \end{array} $$

This formula, however, refers to fermionic and degenerate quantum systems, such as electrons in metals. For ionic systems, such an estimation of kinetic energy is inappropriate because the ion concentration is usually much lower than the concentration of electrons in metals and because the system is not degenerate even if the ions are fermions. The Maxwell-Boltzmann distribution should be applied instead of the Fermi-Dirac or Bose-Einstein distribution. Independent of fermionic or bosonic ion statistics, the Maxwell-Boltzmann distribution allows for an estimation of the averaged kinetic energy of ions located inside a sphere of radius *a* in the following form:

14 $$ <{\Psi}_{ion}|-\sum\limits_{j}\delta(\mathbf{r}-\mathbf{r}_{j})\frac{\hbar^{2}{\nabla_{j}^{2}}}{2m}|{\Psi}_{ion}>\simeq (n+\delta \rho(\mathbf{r},t)){\Theta}(a-r) \frac{3kT}{2}, $$

where *k* is the Boltzmann constant and *T* is the temperature. For ionic molecules with 3D or linear shapes, the inclusion of rotational degrees of freedom results in the factor $\frac {6kT}{2}$ or $\frac {5kT}{2}$, respectively, rather than $\frac {3kT}{2}$ for the point-like ion model.

Using the formula (14) and taking into account that $\nabla {\Theta }(a-r)=-\frac {\mathbf {r}}{r}\delta (a-r)$, Eq. 12 can be rewritten in the following manner:

15 $$\begin{array}{@{}rcl@{}} \frac{\partial^{2} \delta \tilde{\rho} (\mathbf{r},t) }{\partial t^{2}}&=&\left[ \frac{kT}{m}\nabla^{2} \delta \tilde{\rho}(\mathbf{r},t)- {\omega_{p}^{2}} \delta \tilde{\rho}(\mathbf{r},t)\right]{\Theta}(a-r)\\ &&- \frac{kT}{m} \nabla\left\{\left[n+\delta \tilde{\rho}(\mathbf{r},t)\right]\frac{\mathbf{r}}{r}\delta(a-r) \right\}\\ &&-\left[\frac{kT}{m}\frac{\mathbf{r}}{r}\nabla \delta \tilde{\rho}(\mathbf{r},t) + \frac{{\omega_{p}^{2}}}{4\pi} \frac{\mathbf{r}}{r}\nabla \int d^{3}r_{1} \frac{1}{|\mathbf{r}-\mathbf{r}_{1}|} \delta \tilde{\rho}(\mathbf{r}_{1} ,t)\right]\\ &&\times\delta(a-r). \end{array} $$

In this formula, *ω*_*p*_ is the bulk ion-plasmon frequency, ${\omega _{p}^{2}}=\frac {4\pi n q^{\prime 2}}{m}$. The solution of Eq. 15 can be decomposed into two components related to the distinct domains inside the sphere and on the sphere surface:

16 $$ \delta \tilde{\rho}(\mathbf{r,t})=\left\{ \begin{array}{l} \delta \tilde{\rho}_{1}(\mathbf{r,t}), \;for\; r<a,\\ \delta \tilde{\rho}_{2}(\mathbf{r,t}), \;for\; r\geq a,\; (r\rightarrow a+),\\ \end{array} \right. $$

The domains correspond to volume and surface excitations, respectively. These two parts of local ion density fluctuations satisfy the equations (according to Eq. 15),

17 $$ \frac{\partial^{2} \delta \tilde{\rho}_{1} (\mathbf{r},t) }{\partial t^{2}}=\frac{kT}{m} \nabla^{2} \delta \tilde{\rho}_{1}(\mathbf{r},t)- {\omega_{p}^{2}} \delta \tilde{\rho}_{1}(\mathbf{r},t), $$

and (here *𝜖* = 0+),

18 $$\begin{array}{ll} \frac{\partial^{2} \delta \tilde{\rho}_{2} (\mathbf{r},t) }{\partial t^{2}}& =- \frac{kT}{m} \nabla\left\{\left[ n+\delta \tilde{\rho}_{2}(\mathbf{r},t)\right]\frac{\mathbf{r}}{r}\delta(a+\epsilon -r)\right\}\\ & - \left[\frac{kT}{m} \frac{\mathbf{r}}{r}\nabla \delta \tilde{\rho}_{2}(\mathbf{r},t) + \frac{{\omega_{p}^{2}}}{4\pi} \frac{\mathbf{r}}{r}\nabla \int d^{3}r_{1} \frac{1}{|\mathbf{r}-\mathbf{r}_{1}|} \left( \delta \tilde{\rho}_{1}(\mathbf{r}_{1} ,t) {\Theta}(a-r_{1}) \right.\right. \\ & \left.\left. +\delta \tilde{\rho}_{2}(\mathbf{r}_{1} ,t){\Theta}(r_{1}-a)\right)\phantom{\frac{{\omega_{p}^{2}}}{4\pi}}{}\right]\delta(a+\epsilon-r).\\ \end{array}  $$

The Dirac delta in Eq. 18 results from the derivative of the Heaviside step function, the ideal jellium charge distribution. In Eq. 18, an infinitesimal shift, *𝜖* = 0+, is introduced to fulfill the requirements of the Dirac delta definition (its singular point must be an inner point of an open subset of the domain). This shift is only of a formal character and does not reflect any asymmetry.

The electric field due to surface charges is zero inside the sphere and therefore cannot influence the volume excitations. Conversely, the volume charge fluctuation-induced electric field can excite surface fluctuations. Therefore, the equation for volume plasmons is independent of surface plasmons, whereas the volume plasmons contribute to the equation for the surface plasmons.

The problem of separation between surface and volume plasmons has been thoroughly analyzed for metal clusters and has been identified as particularly significant for very small clusters. In the size scale of 1–3 nm for metallic clusters, the effect of so-called electron spill-out beyond the jellium edge is important and causes a fuzzy surface resulting in the coupling of volume and surface plasmon oscillations. Direct numerical simulations of time-dependent local density approximation (TDLDA) [34, 35] have verified that the volume–surface excitation mishmash gradually disappears in larger metallic clusters [34, 35], which supports the accuracy of the semiclassical RPA description, within which volume plasmons are separated from surface ones. The role of spill-out effect diminishes gradually with growing sphere size as the ratio of surface to volume falls down and spill-out becomes negligible in the range of several nanometers for metals and similarly for large ionic spheres. Moreover, for the electrolyte system confined by the insulating membrane, the spill-out of ions is irrelevant. Therefore, the RPA description of ionic density fluctuations is a proper model. Moreover, the analytical RPA semiclassical picture in the form of an oscillator equation allows for convenient inclusion of damping effects, which is especially important since the plasmon damping caused by irradiation losses turns out to be an overwhelming physical property of plasmons in the case of large metallic nanospheres [7, 33] (with *a*>15 nm for Au or Ag) as well as of large ionic systems, as demonstrated in the paragraph “Damping of Plasmon Oscillations in Ionic Systems.”

### Solution of RPA Equations: Volume and Surface Ionic Plasmon Frequencies

**Fig. 1:**
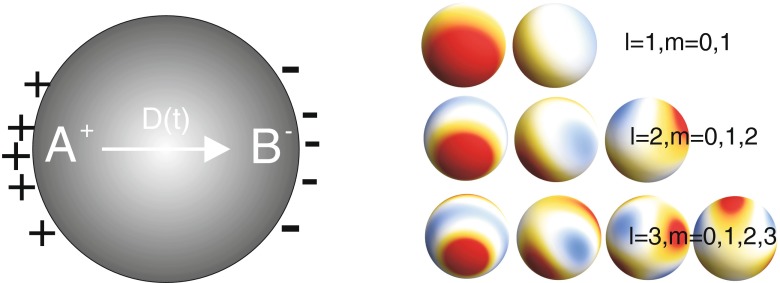
Dipole **D** **(** **t** **)** creation in a single sphere by the simplest surface plasmon oscillations (*left*); examples of surface plasmon charge distribution with various multiplicity *l*, *m*, different colors indicate distinct values of local charge density from *negative-red*to *positive-blue* (*right*)

**Fig. 2:**
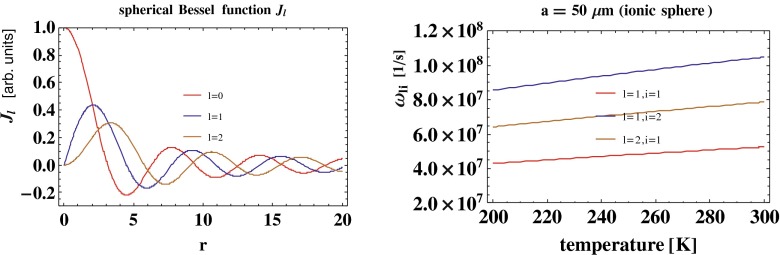
The spherical Bessel functions *J*_*l*_(*r*) for *l* = 0,1,2 displaying possible charge density fluctuations in the sphere along the radius *r* for volume plasmon modes. The angular distribution of these modes is governed by the real spherical functions *Y*_*lm*_(Ω), similar to the surface plasmon modes (cf. Fig. 1 right). *Right*: the exemplary temperature dependence of the self-frequencies of the volume plasmon modes *ω*_*li*_, *l* *i* = 11, 12, 21 for a dilute electrolyte *n*≃10^14^ 1/m ^3^ and ion mass ∼10^4^ *m*_*e*_, *a*∼50μm

Equations 17 and 18 are solved for metallic nanospheres [33], and these solutions can be directly applied to ionic systems. To briefly summarize this analysis, we represent both parts of the plasma fluctuation as follows:

19 $$\begin{array}{@{}rcl@{}} \delta \tilde{\rho}_{1}(\mathbf{r,t})&=&nF(\mathbf{r}, t), \;for\; r<a,\\ \delta \tilde{\rho}_{2}(\mathbf{r,t})&=&\sigma({\Omega},t)\delta(r+\epsilon -a),\\\epsilon&=&0+, \;for\; r\geq a,\; (r\rightarrow a+), \end{array} $$

with initial conditions *F*(**r**, *t*)|_*t* = 0_ = 0, *σ*(Ω, *t*)|_*t* = 0_ = 0, (Ω is the spherical angle), *F*(**r**, *t*)|_*r* = *a*_ = 0, $\int \rho (\mathbf {r},t)d^{3}r=N$ (neutrality condition). With the above initial and boundary conditions and taking advantage of the spherical symmetry, we write the time-dependent parts of the ion concentration fluctuations in the form [33] (cf. Appendix):

20 $$ F(\mathbf{r}, t) =\sum\limits_{l=1}^{\infty}\sum\limits_{m=-l}^{l}\sum\limits_{i=1}^{\infty}A_{lmn}j_{l}(k_{nl}r)Y_{lm} ({\Omega})sin(\omega_{li}t),  $$

and

21 $$\begin{array}{@{}rcl@{}} \sigma({\Omega},t) &=& \sum\limits_{l=1}^{\infty}\sum\limits_{m=-l}^{l} \frac{B_{lm}}{a^{2}}Y_{lm}({\Omega})sin(\omega_{0l}t)\\ &&+ \sum\limits_{l=1}^{\infty}\sum\limits_{m=-l}^{l}\sum\limits_{i=1}^{\infty} A_{lmn}\frac{(l+1){\omega_{p}^{2}}}{l{\omega_{p}^{2}}-(2l+1)\omega_{li}^{2}}Y_{lm}({\Omega})n_{e}\\ &&\times{\int\limits_{0}^{a}} dr_{1} \frac{r_{1}^{l+2}}{a^{l+2}}j_{l}(k_{li}r_{1})sin(\omega_{li}t), \end{array} $$

where $j_{l}(\xi )=\sqrt {\frac {\pi }{2\xi }}I_{l+1/2}(\xi )$ is the spherical Bessel function, *Y*_*lm*_(Ω) is the spherical function (some examples are presented in Fig. 1), $\omega _{li}=\omega _{p}\sqrt {1+\frac {kT x_{li}^{2}}{{\omega _{p}^{2}}a^{2}m}}$ are the frequencies of the ion volume self-oscillations (volume plasmon frequencies), *x*_*li*_ are the nodes of the Bessel function *j*_*l*_(*ξ*) with *i* = 1,2,3… (cf. Fig. 2), *k*_*li*_ = *x*_*li*_/*a*, and $\omega _{l0}=\omega _{p}\sqrt {\frac {l}{2l+1}}$ are the frequencies of the ion surface self-oscillations (surface plasmon frequencies). The derivation of the self-frequencies for ionic plasmon oscillations is presented in more detail in the Appendix. The amplitudes *A*_*lmi*_ and *B*_*lm*_ are arbitrary in the homogeneous problem and can be adjusted to the initial conditions for the first derivatives of the density fluctuations.

The function *F*(**r**, *t*) describes volume plasmon oscillations, whereas *σ*(Ω, *t*) describes surface plasmon oscillations. We emphasize that the first term in Eq. 21 corresponds to the surface self-oscillations, whereas the second describes the surface oscillations induced by the volume plasmons. The frequencies of the surface self-oscillations are equal to

22 $$ \omega_{0l}=\omega_{p}\sqrt{\frac{l}{2l+1}}, $$

which, for *l* = 1, is a dipole-type surface oscillation frequency, described for metallic nanospheres by Mie [36], $\omega _{01}=\omega _{p}/\sqrt {3}$.

### Ionic Surface Plasmon Frequencies for Nanospheres Embedded in a Dielectric Medium

The influence of dielectric surroundings (generally distinct from the inner dielectric of the ionic system) on plasmons in this system can now be included. Let us assume that ions on the surface (*r* = *a*+, i.e., *r*≥*a*, *r*→*a*) interact with Coulomb forces renormalized by the relative dielectric constant *ε*_1_>1 (distinct from the *ε* of the inner medium). Thus, a small modification of Eq. 18 is in order:

23 $$ \begin{array}{ll} \frac{\partial^{2} \delta \tilde{\rho}_{2} (\mathbf{r}) }{\partial t^{2}} &=- \frac{2}{3m} \nabla\left\{\left[\frac{3}{5}\epsilon_{F} n+\epsilon_{F} \delta \tilde{\rho}_{2}(\mathbf{r},t)\right]\frac{\mathbf{r}}{r}\delta(a+\epsilon -r)\right\}\\ & - \left[\frac{2}{3} \frac{\epsilon_{F}}{m}\frac{\mathbf{r}}{r}\nabla \delta \tilde{\rho}_{2}(\mathbf{r},t) \,+\, \frac{{\omega_{p}^{2}}}{4\pi} \frac{\mathbf{r}}{r}\nabla \int d^{3}r_{1} \frac{1}{|\mathbf{r}-\mathbf{r}_{1}|} \left( \delta \tilde{\rho}_{1}(\mathbf{r}_{1} ,t) {\Theta}(a-r_{1})\right.\right.\\ & \left.\left. +\frac{1}{\varepsilon_{1}}\delta \tilde{\rho}_{2}(\mathbf{r}_{1} ,t){\Theta}(r_{1}-a)\right)\right]\delta(a+\epsilon -r),\\ \end{array}  $$

(note that Eq. 17 is not affected by the outer medium). The solution of Eq. 23 is of the same form as that of Eq. 18, but with renormalized surface plasmon frequencies:

24 $$ \omega_{0l}=\omega_{p}\sqrt{\frac{l}{2l+1}\frac{1}{\varepsilon_{1}}}. $$

## Damping of Plasmon Oscillations in Ionic Systems

The presented above semiclassical RPA treatment of plasmon excitations in finite ion systems does not account for plasmon damping. The damping of plasmon oscillations can be included in a phenomenological manner by the addition of an attenuation term to the plasmon dynamic equations, i.e., the term $-\frac {2}{\tau _{0}}\frac {\partial \delta \rho (\mathbf {r},t)}{\partial t}$ added to the right-hand sides of both Eqs. 17 and 18, taking into account their oscillatory form. The introduced damping ratio $\frac {1}{\tau _{0}}$ accounts for ion scattering losses and can be approximated, in analogy to metallic systems, by the inclusion of energy dissipation caused by its irreversible transformation into heat via various microscopic channels, similar to Ohmic resistivity [37]:

25 $$ \frac{1}{\tau_{0}}\simeq \frac{v}{2\lambda_{b} }+\frac{Cv}{2a},  $$

where *a* is the sphere radius, $v=\sqrt {\frac {3 k T}{m}}$ is the mean velocity of ions, and *λ*_*b*_ is the ion mean free path in the bulk electrolyte material (comprising effects of scattering of ions on other ions, on solvent particles and admixtures). The second term in Eq. 25 accounts for the scattering of ions on the boundary of the finite ionic sphere of radius *a*, where the constant *C* is of the order of unity and reflects the type of scattering of ions by the boundary of the sphere dependent of microscopic particularities of the membrane [37].

To explicitly express a forcing field that moves ions in the system, the inhomogeneous time-dependent term should be added to the homogeneous equations (17) and (18). The forcing field may be a time-dependent electric field (e.g., the electric component of the incident e-m wave which may excite plasmons). Similarly as for metallic nanospheres, the surface plasmon resonant wavelength highly exceeds the system dimension in the case of finite ionic systems and the e-m forcing field is essentially space-homogeneous along the whole sphere. Such a perturbation fulfills the so-called dipole approximation requirements and excites only surface dipole plasmons, i.e., the mode with *l* = 1, which can be described by the function *Q*_1*m*_(*t*) (*l* = 1 and *m* are angular momentum numbers related to the assumed spherical symmetry). The corresponding dynamical equation for the surface plasmons reduced to only mode *Q*_1*m*_(*t*) has the following form:

26 $$\begin{array}{@{}rcl@{}} &~&\!\!\!\!\!\!\!\frac{\partial^{2}Q_{1m}(t)}{\partial t^{2}}+\frac{2}{\tau_{0}}\frac{\partial Q_{1m}(t)}{\partial t}+{\omega_{1}^{2}} Q_{1m}(t)\\ &=&\sqrt{\frac{4\pi}{3}}\frac{qn}{m}\left[E_{z}(t)\delta_{m,0}+\sqrt{2}\left( E_{x}(t)\delta_{m,1} + E_{y}(t)\delta_{m,-1}\right)\right],\\ \end{array} $$

where $\omega _{1}=\frac {\omega _{p}}{\sqrt {3\varepsilon _{1}}}$ (a dipole surface plasmon frequency, *ε*_1_ is the dielectric susceptibility of the system surroundings). Because only *Q*_1*m*_ modes contribute to the plasmon response to the homogeneous electric field, the effective ion density fluctuation has the form [33]

27 $$ \delta \rho(\mathbf{r},t)=\left\{ \begin{array}{l} 0,\;\; r<a,\\ \sum\limits_{m=-1}^{1}Q_{1m}(t)Y_{1m}({\Omega})\; r\geq a,\; r\rightarrow a+,\\ \end{array} \right.  $$

where *Y*_*lm*_(Ω) is the spherical function with *l* = 1. One can also explicitly calculate the dipole **D**(*t*) corresponding to surface plasmon oscillations given by Eq. 27:

28 $$ \left\{ \begin{array}{ll} D_{x}(t)&= q^{\prime}\int d^{3}r x\delta\rho(\mathbf{r},t)= \frac{\sqrt{2\pi}}{\sqrt{3}}q^{\prime}Q_{1,1}(t)a^{3},\\ D_{y}(t)&= q^{\prime}\int d^{3}r y\delta\rho(\mathbf{r},t)= \frac{\sqrt{2\pi}}{\sqrt{3}}q^{\prime}Q_{1,-1}(t)a^{3},\\ D_{z}(t)&= q^{\prime}\int d^{3}r z\delta\rho(\mathbf{r},t)= \frac{\sqrt{4\pi}}{\sqrt{3}}q^{\prime}Q_{1,0}(t)a^{3}.\\ \end{array}\right. $$

The dipole **D**(*t*) satisfies the equation (from Eq. 26)

29 $$ \left[\frac{\partial^{2}}{\partial t^{2}}+ \frac{2}{\tau_{0}} \frac{\partial}{\partial t} +{\omega_{1}^{2}}\right] \mathbf{D}(t)=\frac{a^{3} 4\pi q^{{\prime}2}n}{3m} \mathbf{E}(t)=\varepsilon a^{3} {\omega_{1}^{2}} \mathbf{E}(t). $$

Noticeably, the dipole (28) scales as the system volume, ∼*a*^3^, indicating that all ions actually contribute to the surface plasmon oscillations. This observation is connected with the fact that the surface modes correspond to uniform translation-type oscillations of ions in the system when the charge of ions inside the sphere is exactly compensated by oppositely signed ions, whereas unbalanced charge density occurs only on the surface, despite all the ions oscillating. For volume plasmons, non-compensated charge density fluctuations are present inside the sphere because volume plasmon modes have compressional character with unbalanced charge fluctuations along the system radius.

**Fig. 3:**
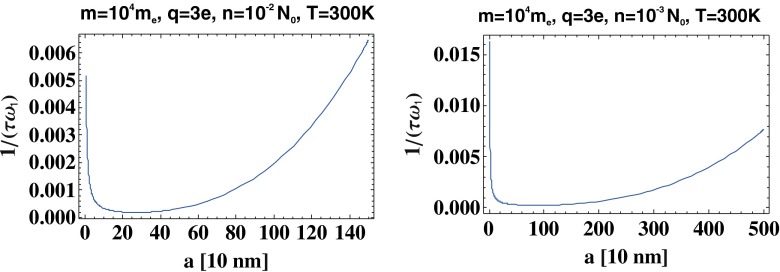
The cross-over in the system size-dependence of the surface plasmon damping rate for *T* = 300 K, *m* = 10^4^ *m*_*e*_, *q* = 3*e*, *n* = 10^−2^ *N*_0_ (where *N*_0_ is the concentration of one molar electrolyte) (*left*) and for *n* = 10^−3^ *N*_0_ (*right*); in the region close to the cross-over, the perturbative treatment for Lorentz friction well coincides with the exact approach

The scattering effects accounted for by the approximate formula (25) cause damping of plasmons and are especially strong for small systems because of the nanosphere-edge scattering contribution, which is proportional to $\frac {1}{a}$. The significance of this term, however, decreases with increasing radius. We will show that radiation losses (due to Lorentz friction) scales initially as *a*^3^ and that, for increasing *a*, these irradiative energy losses quickly dominate plasmon attenuation. Because of the opposite size dependencies of the scattering and irradiation contributions to plasmon damping, we observe a cross-over of damping with respect to the size, as depicted in Fig. 3. In addition, the radius *a*^∗^ for which the total attenuation rate of surface plasmons is minimal, $a^{*}=\left (\frac {9 C v \varepsilon _{1} c^{3}}{2 {\omega _{p}^{4}}}\right )^{1/4}= \left (\frac {9 C \varepsilon _{1} c^{3}}{2 {\omega _{p}^{4}}}\sqrt {3kT/m}\right )^{1/4}$, can also be determined. The system sizes *a*^∗^ for two distinct ionic systems are listed in Table 1.

**Table 1:** The ion system parameters assumed for the calculation of damping rate and self-frequency for dipole surface plasmons

Material	Ionic system	Sample 1	Sample 2
Ion concentration	*n* (*N*_0_ is one-molar concentration)	10^−2^ *N*_0_	10^−3^ *N*_0_
Effective ion mass	*m* (*m*_*e*_ electron mass)	10^4^ *m*_*e*_	10^4^ *m*_*e*_
Effective ion charge	$q/\sqrt {\varepsilon }$	3 *e*	3 *e*
Temperature	*T*	300 K	300 K
Mean velocity of ions	$ v=\sqrt {\frac {3 k T }{ m}}$	1168 m/s	1168 m/s
Bulk plasmon frequency	*ω*_*p*_	9.3×10^13^ 1/s	2.93×10^12^ 1/s
Dielectric constant of surroundings	*ε*_1_	2	2
Mie frequency	$\omega _{1}=\omega _{p}/\sqrt {3\varepsilon _{1}}$	3.8×10^13^ 1/s	1.2×10^12^ 1/s
Constant in Eq. 25	*C*	2	2
Bulk mean free path (room temp.)	*λ*_*b*_	0.1 μm	0.3 μm
Radius for minimal damping	$a^{*}=\left (\frac {9 \varepsilon _{1} Cc^{3}v}{2{\omega _{p}^{3}}}\right )^{1/4}$	2.7×10^−7^ m	8.6×10^−7^ m
Radius for maximal damping	*a* ^∗∗^ from maximum of *I* *m*Ω_2_ given by Eq. 36	8×10^−6^ m	25×10^−6^ m

The radiative energy loss of the oscillating dipole is expressed by the Lorentz friction [17], i.e., the effective electric field slowing the motion of charges:

30 $$ \mathbf{E}_{L}=\frac{2}{3 c^{3}}\frac{\partial^{3}\mathbf{D(t)}}{\partial t^{3}}. $$

Hence, we can rewrite Eq. 29 to include the Lorentz friction term:

31 $$ \left[\frac{\partial^{2}}{\partial t^{2}}+ \frac{2}{\tau_{0}} \frac{\partial}{\partial t} +{\omega_{1}^{2}}\right] \mathbf{D}(t)=\varepsilon a^{3} {\omega_{1}^{2}} \mathbf{E}(t)+ \varepsilon a^{3} {\omega_{1}^{2}} \mathbf{E}_{L}, $$

or for **E** = 0,

32 $$ \left[\frac{\partial^{2}}{\partial t^{2}} +{\omega_{1}^{2}}\right] \mathbf{D}(t)= \frac{\partial}{\partial t}\left[ -\frac{2}{\tau_{0}} \mathbf{D}(t) + \frac{2}{3\omega_{1} \sqrt{\varepsilon_{1}}}\left( \frac{\omega_{p} a}{c\sqrt{3}}\right)^{3} \frac{\partial^{2}}{\partial t^{2}} \mathbf{D}(t) \right]. $$

The perturbation method can be applied for a solution of Eq. 32 when the right-hand side of this equation is treated as a small perturbation. In the zeroth step of the perturbation, we have $\left [\frac {\partial ^{2}}{\partial t^{2}} +{\omega _{1}^{2}}\right ] \mathbf {D}(t)= 0$, from which $ \frac {\partial ^{2}}{\partial t^{2}} \mathbf {D}(t) = -{\omega _{1}^{2}} \mathbf {D}(t)$. Hence, for the first step of the perturbation, we substitute the latter formula into the right-hand side of Eq. 32, i.e.,

33 $$ \left[\frac{\partial^{2}}{\partial t^{2}}+ \frac{2}{\tau} \frac{\partial}{\partial t} +{\omega_{1}^{2}}\right] \mathbf{D}(t)= 0, $$

where

34 $$ \frac{1}{\tau}=\frac{1}{\tau_{0}} +\frac{\omega_{1}}{3\sqrt{\varepsilon_{1}}}\left( \frac{\omega_{p} a}{c\sqrt{3}}\right)^{3}. $$

Within the first step of perturbation, the Lorentz friction can be included in the total attenuation rate $\frac {1}{\tau }$. Nevertheless, this approximation is justified only for sufficiently small perturbations, i.e., when the second term in Eq. 34, which is proportional to *a*^3^, is sufficiently small to fulfill the perturbation restrictions. The related limiting value, $\tilde {a}$, of the ionic system size depends on the ion concentration, charge, mass, and dielectric susceptibility, as is exemplified in the following paragraph.

The solution of Eq. 33 is of the form $\mathbf {D}(t)= \mathbf {A} e^{-t/\tau }cos(\omega _{1}^{\prime } t + \phi )$, where $\omega _{1}^{\prime } =\omega _{1}^{\prime }\sqrt {1-\frac {1}{(\omega _{1}\tau )^{2}}}$ and gives a red shift to the plasmon resonance because of a strong (∼*a*^3^) increase of attenuation caused by the irradiation. The Lorentz friction term in Eq. 34 dominates the plasmon damping for $a^{*}<a<\tilde {a}$ because of this *a*^3^ dependence (cf. Fig. 3). Plasmon damping grows rapidly with *a*, which results in the pronounced red shift of the resonance frequency.

### Exact Inclusion of Lorentz Damping to the Attenuation of Ionic Dipole Surface Plasmons

Let us now consider the dynamic equation for surface plasmons in an ionic spherical system, Eq. 32, with the Lorentz friction term, but without applying the perturbation method for a solution. To compare various contributions to Eq. 32, we change to a dimensionless variable *t*→*t*^′^ = *ω*_1_*t*. Equation 32 becomes the form,

35 $$ \frac{\partial^{2} \mathbf{D}(t^{\prime})}{\partial {t^{\prime}}^{2}}+\frac{2}{\tau_{0} \omega_{1}} \frac{\partial \mathbf{D}(t^{\prime})}{\partial t^{\prime}}+\mathbf{D}(t^{\prime})=\frac{2}{3\sqrt{\varepsilon_{1}}}\left( \frac{\omega_{p} a}{v\sqrt{3}}\right)^{3}\frac{\partial^{3} \mathbf{D}(t^{\prime})}{\partial {t^{\prime}}^{3}}. $$

When solving Eq. 35 by perturbation, we obtain a renormalized attenuation rate for an effective damping term, $\frac {1}{\omega _{1} \tau _{0}}+\frac {1}{3\sqrt {\varepsilon _{1}}}\left (\frac {\omega _{p} a}{v \sqrt {3}}\right )^{3}$. This term quickly reaches unity, for which the oscillator falls into the over-damped regime. For the system parameters assumed for Fig. 3, the attenuation rate reaches unity at 25.5 and 8 μm for *n* = 10^−3^*N*_0_ and *n* = 10^−2^*N*_0_, respectively. At these values of *a*, the frequency $\omega _{1}^{\prime } =\omega _{1}^{\prime }\sqrt {1-\frac {1}{(\omega _{1}\tau )^{2}}}$ goes to zero, which indicates an apparent artifact of the perturbation method. To verify the exact damped frequency behavior in this system, one must solve the dynamical equation (35) without any approximations. As this equation is a third-order linear differential equation, its solution takes the form ∼*e*^*i*Ω*t*′^, with analytical expressions for three possible values of the exponent:

36 $$ \begin{array}{l} {\Omega}_{1} =-\frac{i}{3g}-\frac{i 2^{1/3}(1+6 gu)}{3g{\mathcal{A}}^{1/3}} -\frac{i {\mathcal{A}}^{1/3}}{ 3 \times 2^{1/3}g} \in Im (=i \alpha ),\\ {\Omega}_{2}=- \frac{i}{3g}+\frac{i(1+ i\sqrt{3})(1+6 gu)}{ 3\times 2^{2/3} g {\mathcal{A}}^{1/3}} +\frac{i(1-i\sqrt{3}) {\mathcal{A}}^{1/3}}{ 6 \times 2^{1/3}g}= \omega + i \frac{1}{\tau },\\ {\Omega}_{3}=- \frac{i}{3g}+\frac{i(1- i\sqrt{3})(1+6 gu)}{ 3\times 2^{2/3} g {\mathcal{A}}^{1/3}} +\frac{i(1+i\sqrt{3}) {\mathcal{A}}^{1/3}}{ 6 \times 2^{1/3}g}=-\omega+i \frac{1}{\tau },\\ \end{array} $$

where ${\mathcal {A}}=2+27 g^{2}+18 gu + \sqrt {4(-1-6 gu)^{3}+(2+27 g^{2}+18 gu)^{2}}$, $u = \frac {1}{\tau _{0} \omega _{1}}$ and $g = \frac {2}{3\sqrt {\varepsilon _{1}}} \left (\frac {a\omega _{p}}{c\sqrt {3}}\right )^{3}$.

**Fig. 4:**
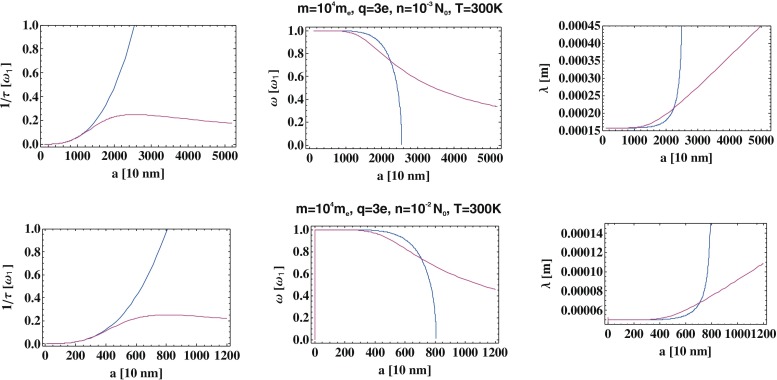
Comparison of the damping rate and the resonance frequency (the latter expressed in the right panel by the resonance wavelength), i.e., the damping rate and frequency (wavelength) of the oscillating solution of Eq. 35, exact one (given by Eq. 36—*red line*) and perturbation approximation (given by Eq. 34—*blue line*), both with respect to the finite ionic system radius *a*

In Fig. 4, we have plotted the damping rate (*Im*Ω_2_) and the self-frequency (*Re*Ω_2_, in right panel the corresponding wavelength is visualized) with respect to the system radius *a*. For comparison, the approximate perturbative solutions are also plotted (in blue line whereas the exact solution of Eq. 35 in red line). The blue line ends at *a*_*limit*_ when the attenuation rate within the perturbation approach reaches the critical value 1 (then *λ*→*∞*). For an accurate solution of Eq. 35, this singular behavior disappears and the oscillating solution exists for larger *a* as well.

We note that the red shift of the plasmon resonance is strongly overestimated in the framework of the perturbative approach to Lorentz friction unless $a<\tilde {a}$, where $\tilde {a}$ is sensitive to ionic system parameters and especially to the ion concentration (demonstrated in Fig. 4).

We emphasize that the Eq. 35 has, in general, two types of particular solutions: *e*^*i*Ω*t*′^, with complex self-frequencies Ω. The solutions given by Ω_2_ and Ω_3_ are of the damped oscillator type (*i*Ω_2_ and *i*Ω_3_ are mutually conjugated, thus Ω_2_ and Ω_3_ have real parts of opposite sign and the same imaginary parts, with the latter being positive and displaying the damping rate), whereas that given by Ω_1_ is an unstable and exponentially rising solution (negative imaginary solution). This unstable solution is a well-known artifact in Maxwell electrodynamics (cf. e.g., $ 75 in [17]) and corresponds to infinite self-acceleration of the free charge due to the Lorentz friction force (i.e., the singular solution of the equation $m\dot {\mathbf {v}}=const. \times \ddot {\mathbf {v}}$, which is associated with a formal renormalization of the field-mass of the charge: infinite for a point-like charge and canceled in an artificial manner by an arbitrarily assumed negative infinite non-field mass, resulting in the ordinary mass of, e.g., an electron, although not defined in a properly mathematical way). This unphysical singular particular solution (Ω_1_) should thus be discarded. The other oscillatory solution resembles that of the ordinary damped harmonic oscillator, though with a distinct attenuation rate and frequency. This attenuation rate and frequency are expressed by analytical formulae for Ω_2_ (or equivalently, Ω_3_) by Eq. 36 and are calculated for various values of *a* and compared with the corresponding quantities found within the perturbation approach. This comparison is presented in Fig. 4. This comparison reveals that the application of the perturbation approach leads to a high overestimation of the damping rate for $a>\tilde {a}$. Therefore, we conclude that the use of the approximate formula for Lorentz friction damping in Eq. 34 is justified up to $a \simeq \tilde {a}$, whereas for $a>\tilde {a}$, this approximate expression causes a large error, rapidly growing with *a*, in comparison to the exact solution. The value $\tilde {a}<a_{limit}$ sharply depends on ionic system parameters and can be approximated by $\tilde {a}\simeq \frac {a_{limit}}{2}$, where *a*_*limit*_ denotes here the sphere radius at which the oscillatory perturbation solution terminates (cf. Fig. 4).

It must be emphasized, however, that in the electrolyte systems there occur additional excitations of the system components which may mix with plasmon oscillations and even could wash out plasmonic effects in similar time scale of oscillations. The most pronounced such an effect is the strong absorption of microwave e-m radiation by rotational degrees of freedom of water dipole molecules. In wide region of GHz, water molecules in liquids and in other materials heavily absorb energy, rotate, and dissipate it next as the heat to the surroundings (for utilization of this effect in microwave ovens typically is used 2.5 GHz e-m wave). Besides of rotational excitations, a separate water molecule exhibits the vibration resonant absorption at ca. 100 THz in vapor phase, whereas in a liquid phase at ca. 50 THz. Therefore, the plasmon frequencies of GHz order and of several THz order frequencies inconveniently are placed in the region of water absorption: the micro-wave rotational one at wide range between 1 and 300 GHz and the vibrational one at ca. 50 THz. To avoid the overlap between plasmon frequencies and absorption frequencies of water, one can address the soft plasmonics of water electrolytes toward lower (of MHz range frequencies) via reduction of the ion concentration and taking advantage of significant increase of water dielectric permittivity in MHz range, lowering plasmon frequency (the relative permittivity of water is *ε*≃80 for frequencies in the MHz range [38], although for higher frequencies, beginning at approximately 10 GHz, this value decreases to approximately 1.7, corresponding to the optical refractive index of water, $\eta \simeq \sqrt {\varepsilon _{1}}=1.33$). Moreover, in non-spherical geometry of an elongate cylinder or strongly prolate ellipsoid, the lowering of longitudinal surface plasmon frequency might be very effective [39, 40], and this frequency can be pushed below the inconvenient GHz window for even larger ion concentrations. Note that such a situation can occur in biological electrolyte systems, e.g., in thin and long electrolyte neuron cords [30].

The developed above model of plasmons allowed for description of plasmon-polaritons in ionic micro-chains of periodically confined electrolytes which has been next utilized to elucidate of previously not known and long-searched mechanism of so-called saltatory conduction in myelinated axons in peripheral nervous system and in the white substance of central nervous system. The transfer of action potential in long axons covered with white lipid myelin (and therefore the name of white substance in brain and in spinal cord) thick sheath exhibits much larger velocity (100 times larger) than the diffusive velocity of ions in neuron cytoplasm. The mechanism of this acceleration is not known and according to observed jumps of the signal between neighboring small intervals in myelin layer called as Ranvier nodes, the related behavior has been called as the saltatory conduction. This jumping of the signal across the myelinated sectors of length typically of 100 μm between consecutive Ranvier nodes accelerates the transfer of action potential and causes that so-called firing of the axon is quick, which in turn is essential for communication and functioning of the body. Upon the assumption of the plasmon-polariton mechanism of neuron firing in the case of periodically myelinated axons, a very good coincidence of signal velocity for realistic parameters of neuron cord size, internal cytoplasm electrolyte molarity, and thickness of the myelin sheath can be achieved [30].

## Conclusions

In summary, we can state that in finite ionic systems, one can observe plasmons similar to those in metallic nanoparticles. The structure of ionic surface and volume plasmons is analogous to similar properties of electronic plasmons in metallic spherical systems, albeit with a significant red shift of resonance energy corresponding to the far larger mass of ions compared to that of the electrons and the lower concentration of ions in electrolytes compared to the concentration of electrons in metals. Thus, corresponding to the resonance energy, wavelength is shifted to deep-infrared or even longer wavelengths depending on ion concentration. The typical cross-over in the plasmon damping size dependence for metal clusters between the scattering, Ohmic-type energy dissipation, and the radiative losses is observable in spherical ionic systems. This dependence is similar to the size-dependence in metals, though shifted toward the micrometer scale for ions instead of toward the nanometer scale for metals. Of particular interest is the high irradiation regime for dipole plasmons in ionic systems, with potential applications for signaling and energy transfer. The initial strong enhancement of the efficiency of the Lorentz friction with increasing radius of the electrolyte sphere is observed on the micrometer scale with typical *a*^3^ radius dependence above some threshold that depends on the electrolyte parameters. At a certain value of the radius (which also strongly depends on the ion system parameters), this enhancement saturates and the radiative losses slowly diminish, which allows for the definition of the most convenient size of the finite electrolyte system for optimizing radiation-mediated energy transport efficiency, preferring the highest radiation losses.

## Appendix: Derivation of Plasmon Frequencies

### Volume Plasmons

To determine the self-frequencies of volume ionic plasmons in a sphere, we must solve Eq. 17 in the form of the relevant solution given by Eq. 19. For the initial conditions listed below Eq. 19, we assume *F*(**r**, *t*) = *F*_*ω*_(**r**)*s**i**n*(*ω**t*), and, by substitution of this function into Eq. 17, we obtain,

37 $$ {\Delta} F_{\omega}(\mathbf{r})+k^{2}F_{\omega}(\mathbf{r})=0, $$

where $k^{2}=\frac {(\omega ^{2}-{\omega _{p}^{2}})m}{kT}$. This equation is a well-known Helmholtz differential equation with solutions (finite at the origin) expressed by the spherical Bessel functions (for the radial dependence of *F*_*ω*_(**r**)),

38 $$ F_{\omega}(\mathbf{r})=Aj_{l}(kr)Y_{lm}({\Omega}), $$

where $j_{l}(x)=\sqrt {\frac {\pi }{2x}}I_{l+1/2}(x)$ is the *l*th spherical Bessel function of the first kind (the angle dependence is given by the spherical functions *Y*_*l**m*_(Ω)). The boundary condition, *F*(*a*) = 0, gives quantization of *k*, $k_{li}=\frac {x_{li}}{a}$, where *x*_*l**i*_ is the *i*th zero of the *l*th Bessel function (cf. Fig. 2 left). After this quantization, we arrive at the corresponding self-frequency quantization:

39 $$ \omega_{li}^{2}={\omega_{p}^{2}}\left( 1+\frac{kTx_{li}}{{\omega_{p}^{2}} m a^{2}}\right).  $$

Thus, the volume ionic plasmons in the sphere are described by

40 $$ \delta\rho_{1}(\mathbf{r},t)=n\sum\limits_{l=1}^{\infty}\sum\limits_{m=-l}^{m=l}{\sum}_{i=1}^{\infty}A_{lmi} j_{l}(k_{li}r)Y_{lm}({\Omega})sin(\omega_{li}t), $$

where *A*_*l**m**i*_ are arbitrary constants. The component with *l* = 0 vanishes because of the neutrality condition, ${\int \limits _{0}^{a}}r^{2} dr d{\Omega } F(\mathbf {r},t)=0$ (as $\int d{\Omega } Y_{lm}({\Omega })=\sqrt {4\pi } \delta _{l0}\delta _{m0}$, *d*Ω=*s**i**n*Θ*d*Θ*d**ϕ*). Note that, in ionic systems, self-frequencies of volume plasmons in the sphere are temperature dependent (cf Eq. 39 and Fig. 2 right) contrary to plasmons in metals.

### Surface Plasmons

To determine self-frequencies for surface plasmons, Eq. 18 and its solution given by Eq. 19 must be considered. The first term in the right-hand side of Eq. 18 can be rewritten in the form,

41 $$ \begin{array}{l} \frac{kT}{m}\nabla(n+\delta\rho_{2})\nabla{\Theta}(a-r)+\frac{kT}{m}(n+\delta\rho){\Delta}{\Theta}\\ =-\frac{kT}{m}\delta(a-r)\frac{\partial}{\partial r}(n+\delta\rho)=\frac{kT}{m}\frac{1}{r^{2}}\frac{\partial}{\partial r}(r^{2}\delta(a-r)\\ =-\frac{kT}{m}\frac{1}{r^{2}}\frac{\partial}{\partial r }\left[(n+\delta \rho_{2})r^{2}\delta(a-r)\right], \end{array} $$

where we used $\nabla {\Theta }(a-r)=-\frac {\mathbf {r}}{r}\delta (a-r)$, $\frac {\mathbf {r}}{r} \nabla =\frac {\partial }{\partial r}$. The next term in the right-hand side of Eq. 18 can be transformed into,

42 $$ \begin{array}{l} -\frac{kT}{m}\delta (a-r)\frac{\mathbf{r}}{r}\nabla \delta\rho_{2}-\frac{{\omega^{2}_{p}}}{4\pi}\delta(a-r)\frac{\mathbf{r}}{r}\nabla\int \frac{d^{3}r_{1}\delta\rho(\mathbf{r_{1}})}{|\mathbf{r}-\mathbf{r_{1}}|}\\ =-\frac{kT}{m}\delta(a-r)\frac{\partial }{\partial r}\delta\rho-2- \frac{{\omega^{2}_{p}}}{4\pi}\delta(a-r) \frac{\partial}{\partial r}\int\frac{d^{3}r_{1} \delta\rho(\mathbf{r}_{1})}{|\mathbf{r}-\mathbf{r}_{1}|}. \end{array} $$

Equation 18 thus becomes,

43 $$\begin{array}{@{}rcl@{}} \frac{\partial^{2}\rho_{2}}{\partial t^{2}}&=& -\frac{kT}{m}\frac{1}{r^{2}}\frac{\partial}{\partial r }\left[(n+\delta \rho_{2})r^{2}\delta(a-r)\right]\\ &&-\frac{kT}{m}\delta(a-r)\frac{\partial }{\partial r}\delta\rho-2- \frac{{\omega^{2}_{p}}}{4\pi}\delta(a-r) \frac{\partial}{\partial r}\\ &&\times\int\frac{d^{3}r_{1} \delta\rho(\mathbf{r}_{1})}{|\mathbf{r}-\mathbf{r}_{1}|}. \end{array} $$

We suppose the solution of Eq. 43 to be in the form, *δ**ρ*_2_ = *σ*(Ω, *t*)*δ*(*a*+0^+^−*r*) and multiply both sides of this equation by *r*^2^ and integrate with respect to *r* in arbitrary limits, i.e., ${\int \limits _{l}^{L}}r^{2}dr\dots $, such that *a*∈(*l*, *L*) (in order to remove Dirac deltas). This operation leads to the equation,

44 $$\begin{array}{@{}rcl@{}} a^{2}\frac{\partial^{2} \sigma({\Omega},t)}{\partial t^{2}}&=&-\frac{kT}{m}{\int\limits_{l}^{L}}dr\frac{\partial}{\partial r}\left[ (n+\delta\rho_{2})r^{2}\delta(a-r)\right]\\ &&-\frac{kT}{m}\sigma({\Omega},t){\int\limits_{l}^{L}}r^{2} dr \delta(a-r)\frac{\partial}{\partial r}\delta(a-r)\\ &&-\frac{{\omega_{p}^{2}}}{4\pi} {\int\limits_{l}^{L}}r^{2}dr \delta(a-r)\frac{\partial }{\partial r}\int\limits_{a}^{\infty}{r_{1}^{2}}dr_{1}\int d{\Omega}\frac{\delta\rho_{2}(\mathbf{r}_{1})}{|\mathbf{r} -\mathbf{r}_{1}|}\\ &&-\frac{{\omega_{p}^{2}}}{4\pi} {\int\limits_{l}^{L}}r^{2}dr \delta(a-r)\frac{\partial }{\partial r}{\int\limits_{0}^{a}}{r_{1}^{2}}dr_{1}\int d{\Omega}\frac{\delta\rho_{1}(\mathbf{r}_{1})}{|\mathbf{r} -\mathbf{r}_{1}|}. \end{array} $$

The first two terms in the right-hand side of Eq. 44 vanish because,

45 $$\begin{array}{@{}rcl@{}} &~&\!\!\!\!\!\!\!\!-\frac{kT}{m}{\int\limits_{l}^{L}}dr\frac{\partial}{\partial r}\left[ (n+\delta\rho_{2})r^{2}\delta(a-r)\right]\\ &=&-\frac{kT}{m}\left[(n+\delta\rho_{2})r^{2} \delta(r-a)\right] |_{l}^{L}=0 \end{array} $$

and

46 $$\begin{array}{@{}rcl@{}} &~&\!\!\!\!\!\!\!\!-\frac{kT}{m}\sigma({\Omega},t){\int\limits_{l}^{L}}r^{2} dr \delta(a-r)\frac{\partial}{\partial r}\delta(a-r)\\&=&-\frac{kT}{m}a^{2}{\int\limits_{l}^{L}}dr \frac{1}{2}\frac{\partial}{\partial r}\delta^{2}(a-r)\\ &=&-\frac{kT}{m}\frac{a^{2}}{2}\delta^{2}(a-r)|_{l}^{L}\\&=&-\frac{kT}{m}\frac{a^{2}}{2} lim_{\mu\rightarrow 0}\frac{1}{\pi}\frac{\mu}{\mu^{2}+(a-r)^{2}} \delta(a-r)|_{l}^{L}=0. \end{array} $$

The last two terms of the right-hand side of Eq. 44 can be transformed using the generating function for Legendre polynomials [41],

47 $$ \frac{1}{\sqrt{1+z^{2}-2zcos\gamma}}=\sum\limits_{l=0}^{\infty}P_{l}(cos\gamma)z^{l},\;for\; \;z<1, $$

where $P_{l}(cos\gamma )=\frac {4\pi }{2l+1}\sum \limits _{m=-l}^{l}Y_{lm}({\Omega })Y^{*}_{lm}({\Omega })$ are Legendre polynomials. This formula leads to the following one:

48 $$ \frac{\partial}{\partial a}\frac{1}{|\mathbf{a}-\mathbf{r}_{1}|}=\left\{\begin{array}{l} \sum\limits_{l=0}^{\infty}\frac{la^{l-1}}{r_{1}^{l+1}}P_{l}(cos\gamma),\;for\;a<r_{1},\\ -\sum\limits_{l=0}^{\infty}\frac{(l+1){r_{1}^{l}}}{a^{l+2}} P_{l}(cos\gamma),\;for \;a>r_{1},\\ \end{array}\right.  $$

where $\mathbf {a}=a\frac {\mathbf {r}}{r}$ and $cos\gamma =\frac {\mathbf {a}\cdot \mathbf {r}_{1}}{ar_{1}}$. Employing Eq. 48, the last two terms in Eq. 44 can be transformed as follows:

49 $$ \begin{array}{l} -\frac{{\omega_{p}^{2}}}{4\pi}{\int\limits_{l}^{L}}r^{2}dr\delta(a-r)\frac{\partial}{\partial r}\int\limits_{a}^{\infty}{r_{1}^{2}}dr_{1}\int d{\Omega}_{1}\frac{\delta\rho_{2}(\mathbf{r}_{1})}{|\mathbf{r} -\mathbf{r}_{1}|}\\ =-\frac{{\omega_{p}^{2}}}{4\pi}a^{2}\int d{\Omega}_{1} \int\limits_{a}^{\infty}{r_{1}^{2}}dr_{1}\delta\rho_{2}(\mathbf{r}_{1})\frac{\partial}{\partial a}\frac{1}{\sqrt{a^{2}+{r^{2}_{1}}-2ar_{1}cos\gamma}}\\ =-\frac{{\omega_{p}^{2}}}{4\pi}a^{2}\int d{\Omega}_{1} \int\limits_{a}^{\infty} {r_{1}^{2}}dr_{1} \sigma({\Omega}_{1}) \delta(a+0+-r_{1})\\\times \sum\limits_{l=0}^{\infty}\frac{la^{l-1}}{r_{1}^{l+1}}P_{l}(cos\gamma)\\ =-\frac{{\omega_{p}^{2}}}{4\pi} a^{2} \int d{\Omega}_{1} \sigma ({\Omega}_{1}) \frac{1}{a^{2}} \sum\limits_{l=0}^{\infty} \frac{4\pi l}{2l+1}\sum\limits_{m=-l}^{l} Y_{lm}({\Omega}) Y^{*}_{lm}({\Omega}_{1})\\ =-{\omega_{p}^{2}} a^{2}\sum\limits_{l=0}^{\infty}\sum\limits_{m=-l}^{l}\frac{l}{2l+1}Y_{lm}({\Omega}) \int d{\Omega}_{1}\sigma({\Omega}_{1})Y^{*}_{lm}({\Omega}_{1}),\\ \end{array} $$

and

50 $$ \begin{array}{l} -\frac{{\omega_{p}^{2}}}{4\pi}{\int\limits_{l}^{L}}r^{2}dr\delta(a-r)\frac{\partial}{\partial r}{\int\limits_{0}^{a}}{r_{1}^{2}}dr_{1}\int d{\Omega}_{1}\frac{\delta\rho_{1}(\mathbf{r}_{1})}{|\mathbf{r} -\mathbf{r}_{1}|}\\ =-\frac{{\omega_{p}^{2}}}{4\pi}a^{2}\int d{\Omega}_{1} {\int\limits_{0}^{a}}{r_{1}^{2}}dr_{1} nF(\mathbf{r}_{1},t)(\mathbf{r}_{1})\frac{\partial}{\partial a}\frac{1}{\sqrt{a^{2}+{r^{2}_{1}}-2ar_{1}cos\gamma}}\\ =\frac{{\omega_{p}^{2}}}{4\pi} a^{2} \int d{\Omega}_{1} nF(\mathbf{r_{1}},t) \sum\limits_{l=0}^{\infty} \frac{(l+1){r_{1}^{l}}}{a^{l+2}}P_{l}(cos\gamma)\\ ={\omega_{p}^{2}} n \sum\limits_{l=0}^{\infty} \frac{l+1}{2l+1}Y_{lm}({\Omega}){\int\limits_{0}^{a}}{r_{1}^{2}}dr_{1}\frac{{r_{1}^{l}}}{a^{l}}\sum\limits_{l_{1}=1}^{\infty} \sum\limits_{m_{1}=-l_{1}}^{l_{1}}\\\times\sum\limits_{i}A_{lmi}j_{l_{1}}(k_{l_{1}i}r_{1})sin(\omega_{l_{1}i}t)\int d{\Omega}_{1}Y^{*}_{lm}({\Omega}_{1})Y_{l_{1}m_{1}}({\Omega}_{1})\\ ={\omega_{p}^{2}} n\sum\limits_{l=0}^{\infty}\sum\limits_{m=-l}^{l} \sum\limits_{i}\frac{l+1}{2l+1}Y_{lm}({\Omega}) A_{lmi}\\\times{\int\limits_{0}^{a}}\frac{r_{1}^{l+2} dr_{1}}{a^{l}}j_{l}(k_{li}r_{1})sin(\omega_{li}t).\\ \end{array} $$

Equation 44 thus becomes,

51 $$\begin{array}{@{}rcl@{}} \frac{\partial^{2} \sigma({\Omega},t)}{\partial t^{2}} &=&-{\omega_{p}^{2}} a^{2}\sum\limits_{l=0}^{\infty}\sum\limits_{m=-l}^{l}\frac{l}{2l+1}Y_{lm}({\Omega})\\ &&\times\int d{\Omega}_{1}\sigma({\Omega}_{1})Y^{*}_{lm}({\Omega}_{1})\\ &&+{\omega_{p}^{2}} n\sum\limits_{l=0}^{\infty}\sum\limits_{m=-l}^{l} \sum\limits_{i}\frac{l+1}{2l+1}Y_{lm}({\Omega}) A_{lmi}\\&&\times{\int\limits_{0}^{a}}\frac{r_{1}^{l+2} dr_{1}}{a^{l}}j_{l}(k_{li}r_{1})sin(\omega_{li}t), \end{array} $$

Assuming that $\sigma ({\Omega },t)=\sum \limits _{l=0}^{\infty }\sum \limits _{m=-l}^{l}q_{lm}(t)Y_{lm}({\Omega })$ and substituting it into the above equation, we obtain,

52 $$\begin{array}{@{}rcl@{}} \sum\limits_{l=0}^{\infty}\sum\limits_{m=-l}^{l}Y_{lm}({\Omega})\frac{\partial^{2}q_{lm}(t)}{\partial t^{2}}&=&-\sum\limits_{l=0}^{\infty}{\sum}_{m=-l}^{l} \frac{{\omega_{p}^{2}}l}{2l+1}Y_{lm}({\Omega})q_{lm}(t)\\ &&+{\omega_{p}^{2}}\sum\limits_{l=1}^{\infty}\sum\limits_{m=-l}^{l}\sum\limits_{i} \frac{l+1}{2l+1}Y_{lm}({\Omega})A_{lm}\\&&\times{\int\limits_{0}^{a}}\frac{r_{1}^{l+2}dr_{1}}{a^{l+2}}j_{l}(k_{li}r_{1})sin(\omega_{li}t). \end{array} $$

We note that, for *l* = 0 we obtain $\frac {\partial ^{2}q_{00}}{\partial t^{2}}=0$, thus, *q*_00_(*t*) = 0 (as *q*(0)=0 and $\lim _{t\rightarrow \infty } q(t)<\infty $). For *l*≥1, we obtain,

53 $$\begin{array}{@{}rcl@{}} \frac{\partial^{2} q_{lm}(t)}{\partial t^{2}}&=&-\frac{{\omega_{p}^{2}} l}{2l+1}q_{lm}(t)+\sum\limits_{i}{\omega_{p}^{2}}\frac{l+1}{2l+1} A_{lm} n\\&&\times{\int\limits_{0}^{a}}\frac{r_{1}^{l+2}dr_{1}}{a^{l+2}}j_{l}(k_{li}r_{1})sin(\omega_{li}t), \end{array} $$

which requires a solution of the form,

54 $$\begin{array}{@{}rcl@{}} q_{lm}(t)&=&\frac{B_{lm}}{a^{2}} sin(\omega_{p}\sqrt{\frac{l}{2l+1}}t)\\ &&+\sum\limits_{i} A_{lm}\frac{(l+1){\omega_{p}^{2}}}{{\omega_{p}^{2}}-(2l+1)\omega_{li}^{2}}n\\ &&\times{\int\limits_{0}^{a}}\frac{r_{1}^{l+2}dr_{1}}{a^{l+2}}j_{l}(k_{li}r_{1})sin(\omega_{li}t), \end{array} $$

and $\delta \rho _{2}(\mathbf {r},t)=\sum \limits _{l=1}^{\infty }\sum \limits _{m=-l}^{l}q_{lm}(t)Y_{lm}({\Omega }) \delta (a-r)$. The first term in Eq. 54 describes the self-oscillations of surface plasmons, whereas the second term displays the surface plasmon oscillations induced by the volume plasmons. The volume-plasmon-induced component of the surface oscillations is nonzero only when the volume modes are excited and their amplitudes, *A*_*l**m**i*_, are nonzero. The frequencies of the self-oscillations of the surface plasmons are equal to $\omega _{l0}=\omega _{p}\sqrt {\frac {l}{2l+1}}$, corresponding to various multipole modes (numbered with *l*). Noticeably, these frequencies are lower that the bulk plasmon frequency $\omega _{p}=\sqrt {\frac {n q^{2} 4\pi }{m}}$, whereas the volume plasmon modes oscillate with frequencies greater than *ω*_*p*_ (as $\omega _{li}= \omega _{p}\sqrt {1+\frac {kT x_{li}^{2}}{{\omega _{p}^{2}}a^{2}m}}$). Also noteworthy is the absence of the temperature dependence of the ionic surface plasmon resonances, unlike the volume plasmon self-frequencies.
